# Systematic review of the epidemiology of eating disorders in the Arab world

**DOI:** 10.1097/YCO.0000000000000960

**Published:** 2024-08-16

**Authors:** Bernou Melisse, Eric van Furth, Hans W. Hoek

**Affiliations:** a American Center for Psychiatry and Neurology, Al-Manhal, Abu Dhabi, Abu Dhabi, United Arab Emirates; b Co-Eur, P.O. box 30514. 3503AH, Utrecht, the Netherlands; c Utrecht University, Department of Clinical Psychology, Utrecht; d Tilburg University, Department of Medical and Clinical Psychology, Postbus 90153, 5000 LE Tilburg; e GGZ Rivierduinen Eating Disorders Ursula; f Department of Psychiatry, Leiden University Medical Center, Leiden; g Parnassia Psychiatric Institute the Netherlands, The Hague; h Groningen University, Department of Psychiatry, Groningen, the Netherlands; i Columbia University New York, Department of Epidemiology, Mailman School of Public Health, New York, New York, USA

**Keywords:** anorexia nervosa, Arab world, binge-eating disorder, bulimia nervosa, eating disorders, epidemiology, prevalence

## Abstract

**Purpose of review:**

The Arab world is dealing with modernization and sociocultural changes both associated with eating disorders. The present review provides an update of ‘Eating disorders in the Arab world: a literature review’, which was published in 2020.

**Recent findings:**

There are 22 recent epidemiological studies on eating disorders in five different countries in the Arab world. A large-scale national mental health survey reported a 12-month eating disorder prevalence of 3.2% and an eating disorder lifetime prevalence of 6.1%. Binge-eating disorder was the most common eating disorder (12-month prevalence = 2.1%, lifetime prevalence = 2.6%), 1.6% was at high risk for binge-eating disorder. Overall, between 23.8 and 34.8% was at high risk for any eating disorder. Body-shape dissatisfaction, a high BMI and separated/widowed/single marital status were associated with eating disorder pathology.

**Summary:**

Although there is still a lack of studies compared to the western world, the number of epidemiological studies on eating disorders in the Arab world is growing and there is an increase in studies using appropriate assessment-tools and norms. It is recommended to offer specialized treatment and to implement preventive programs.

## Video Abstract

Video Abstract

## INTRODUCTION

Eating disorders occur across the globe and particularly in cultures in transition, which illuminates the interplay between culture and psychopathology [1–5]. In this journal in 2014, Pike *et al*. [6] stated that eating disorders appear to be increasing in the Arab region also in concert with increasing industrialization, urbanization, and globalization. The tendencies that predispose both men and women to developing an eating disorder, namely unhealthy dieting practices, restrictive eating, body preferences rooted in the ‘thin ideal’, bodyweight and body-shape dissatisfaction, and weight misperception, are increasing in many parts of the Arab region [6]. Evidence from some Arab countries is also indicative of an increase in subclinical and clinical eating disorders. For example, in 2010, one-third of Jordanian girls (*N* = 432) between the age of 10 and 16 years reported significant eating abnormality, with prevalence estimates of 0.6% for bulimia nervosa, 1.8% for binge-eating disorder (BED), and 31% for eating disorder not otherwise specified (EDNOS) [7]. It is notable that the study did not find any cases of anorexia nervosa.

A more recent review, involving Arab Middle Eastern countries of the Eastern Mediterranean Region [8], found large variations in the range of the population being at high risk for an eating disorder and also identified various factors associated with being at high risk for an eating disorder and disturbed eating behaviors. Arab societies dealing with rapid industrialization, globalization, and westernization were more at risk for an eating disorder and reported elevated prevalences of disturbed eating behaviors [9]. Currently, the Arab world, particularly the Gulf region (Bahrain, Kuwait, Oman, Saudi Arabia, Qatar, United Arab Emirates [UAE]) [10], is still dealing with rapid industrialization, globalization, and becoming more progressive. Since 2019, significant changes have occurred aimed advancing societal modernization. For instance, women in Saudi Arabia now can drive and travel without the permission of a guardian, and there has been relaxation of the mandatory abaya and veil [11]. In 2024, Saudi Arabia joins the Miss Universe competition for the first time [12]. Additionally, in the UAE, women have the right to undergo emergency abortions without needing their husband's consent [13]. Moreover, unmarried couples in the UAE are allowed to receive in-vitro fertilization treatments, can live together, and there is no longer a strict requirement for marriages to adhere solely to Sharia law. Furthermore, divorce procedures have been streamlined in both Saudi Arabia and the UAE [11,13]. Furthermore, the Gulf area has modernized with regards to leisure as well. Saudi Arabia only used to allow work visas, now offers tourist visas [11], concerts are allowed [14], and some Gulf countries such as Saudi Arabia, Qatar, and the UAE heavily invested in culture [15], for example, various museums and cultural sites have been opened or are currently build. Finally, regulations around alcohol use have been relaxed in Saudi Arabia, Qatar, and the UAE [14,16].

The rapid transformations are leading to various emerging factors associated with an increased risk for eating disorders [9]. Adolescents, in particular, are vulnerable, and currently, 25–50% of the Arab Gulf population is aged under 25 [17]. Additionally, the shift towards a more sedentary lifestyle correlates with rising BMI (kg/m^2^), alongside an amplification of the ‘thin ideal’. Consequently, approximately 70% of the population has a high BMI (BMI > 25) [17,18^▪^], and the interaction between increased BMI and the ‘thin ideal’ contributes to heightened levels of body-shape disstatisfaction [9]. Moreover, an escalation in socioeconomic status is linked to a heightened risk of eating disorders, with several Gulf countries ranking among those with the highest Gross Domestic Product levels and offering tuition-free universities [19].

The ongoing sociocultural changes in the Arab world might be associated with a further increase in the number of individuals at high risk for an eating disorder. Therefore, an update is provided on systematic review published in 2020 [9]. The aim of the present study is to provide estimates of the prevalence of eating disorders, individuals at high risk for an eating disorder, and disturbed eating behaviors in the Arab world. The present review covers peer-reviewed studies from August 2019 until April 2024. 

**Box 1:**
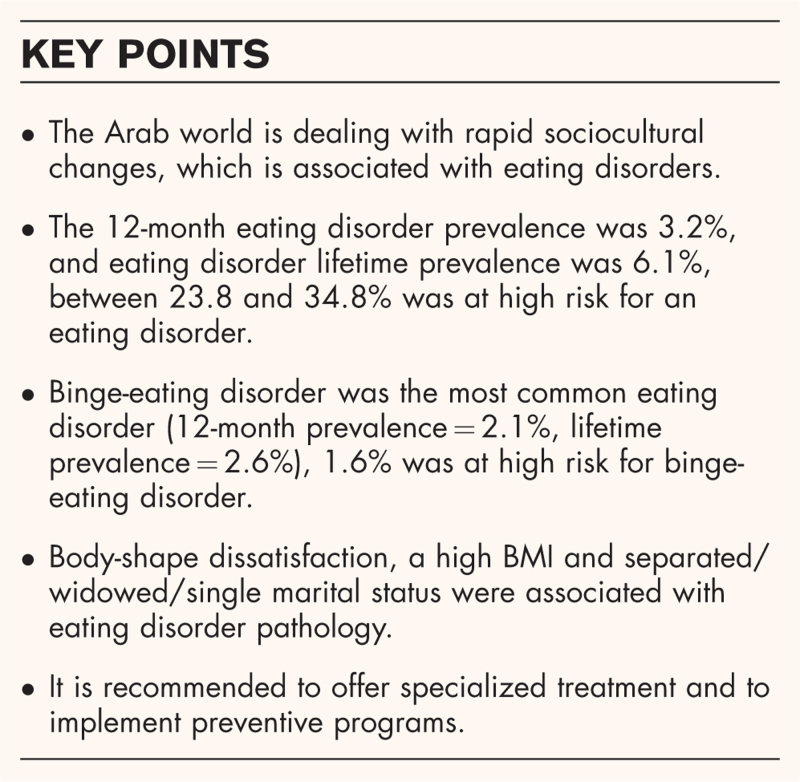
no caption available

## MATERIALS AND METHODS

### Search strategy and study selection

Peer-reviewed studies published in English were included in the present systematic review, regardless of study design in order to cover all relevant available studies to date. Literature was systematically searched and reviewed from Web of Science, PubMed (Medline), PsycINFO, ScienceDirect, WorldCat, and Google Scholar. Since the present review was an update of a review published in 2020 [9], time window was August 2019 up to April 2024. The search aimed to find studies that reported on eating disorders, a high risk for eating disorders, and eating disorder behaviors, such as binge eating and restraint eating, in the Arab world.

Combinations of eating disorder terms and the names of Arab countries were searched. Moreover, to identify further suitable studies, reference lists of included publications were searched manually. Subsequently, identified studies were imported into Endnote, and deduplicated. As an initial step, the first author performed a content assessment based on study titles. Consequently, abstracts and key words were screened for eligibility. Eligible studies were conducted in Arab countries in the Middle East, providing estimates regarding the point prevalence of eating disorders, individuals at high risk for eating disorders, and eating disorder behaviors.

### Inclusion and exclusion criteria

Studies had to meet the following criteria for inclusion: they had to be conducted in Arab Middle Eastern countries of the Eastern Mediterranean Region [8], which includes West Asian countries (Iraq, Jordan, Lebanon, Syria), the Gulf (Bahrain, Kuwait, Qatar, UAE, Saudi Arabia, Oman), and Palestinian populations [8], they included eating disorders, individuals at high-risk, or eating disorder behaviors, and they reported prevalence rates. Studies were excluded when they reported about North African countries such as Morocco, when they included non-Arab Middle Eastern countries situated in the Eastern Mediterranean Region, such as Iran, studies that solely included high BMI, and qualitative studies without empirical data. Due to the paucity of relevant studies, there were no restrictions on age or sex.

### Quality assessment, publication bias, and data abstraction

**FIGURE 1:**
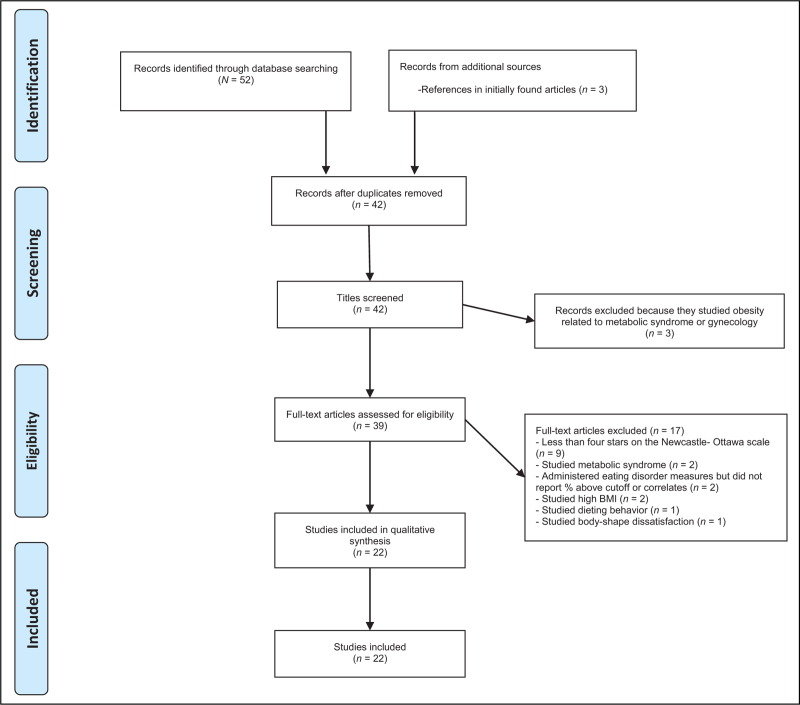
Flow chart of the study selection process based on PRISMA.

The Newcastle-Ottawa scale [20], a seven-item scale that investigates the risk of bias, power, research design, sample, recruitment, and statistical analysis was used to evaluate the quality of the included studies. Appendix A, A shows that studies could obtain a maximum score of 10 stars, which was based on selection (a maximum five stars), comparability (a maximum two stars), and outcome (a maximum three stars). In accordance with a previous review conducted [9,21], studies with at least four stars were included in the present review. Subsequently, study selection was in accordance with the Preferred Reporting Items for Systematic Reviews and Meta-Analyses (PRISMA) guidelines (Fig. 1) [22].

### Assessment of eating disorders, at high risk for eating disorders, disturbed eating behaviors

Semi-structured diagnostic interviews are designed to diagnose eating disorders, and preferred above self-reports to identify eating disorder behaviors such as binge-eating episodes, purging behavior [21,22]. Furthermore, diagnostic concordance between diagnostic interviews and self-report is moderate at best [23]. The reports of eating disorder behaviors such as binge-eating, and purging behavior tend to diverge between self-reports and investigator-based assessments [24]. However, individuals at high risk of an eating disorder, as well as the severity of eating disorder pathology and body-shape dissatisfaction may be accurately measured by self-report [21].

## RESULTS

Of the 22 studies included (Fig. 1), 14 were of cross-sectional nature, and 8 were validation studies. Table 1 summarizes that most studies were conducted in Lebanon (*n* = 13), followed by Saudi Arabia (*n* = 5). No relevant studies were identified in Iraq, Jordan, Kuwait, Syria, UAE, and Qatar. No studies were identified that reported about clinical samples.

**Table 1:** Summary of studies reporting the prevalence of eating disorders, being at high risk for an eating disorder, and disturbed eating behaviors, as well as correlates of eating disorder pathology in Arab countries of the Eastern Mediterranean region

Country/ population	Ref.	Participants, sex, study design	Measures	Outcomes
Bahrain	Abdulla *et al.*[31^▪^]	*N *= 959, Bahraini citizens, aged 15–30 years, CS	BES, PHQ-9	At high risk for binge eating = 21.2%. Binge eating was positively associated with BMI, dietary restraint, depressive symptoms, and anxiety.
Kuwait	Ebrahim *et al.*[44]	*N *= 400, Kuwaiti undergraduate students, male, age range not mentioned, CS	EAT, BIG	At high risk for an eating disorder = 46.2%, at high risk for clinical body-shape dissatisfaction = 63–67, being at high risk for an eating disorder was positively associated with body-shape dissatisfaction and BMI.
Lebanon	Saade *et al.*[36]	*N *= 811, Lebanese citizens, aged > 18 years, CS	DRES, EES, RSES, BDS- EDI-2, HAM-D	At high risk for an eating disorder = 48.3%, being at high risk for an eating disorder was positively associated with being female, age, body-shape dissatisfaction, BMI, exercising, experienced media pressure, anxiety.
Lebanon^a^	Zeidan *et al.*[35]	*N *= 811, Lebanese citizens, aged > 18 years, VS	BES, DRES, EES, RSES, BDS- EDI-2, HAM-D	Binge eating was positively associated with emotional eating, body-shape dissatisfaction, BMI, experienced media pressure, anxiety, depression.
Lebanon^a^	Boulos Nakhoul *et al.*[38]	*N *= 555, Lebanese citizens, aged 15--18 years, VS	DRES, BDS- EDI-2, RSES, PHQ-9, BDS	Being at risk for an eating disorder, BMI, body-shape disstatisfaction were positively associated with each other.
Lebanon^a^	Deek *et al.*[45^▪^]	*N *= 377, Lebanese female university students, aged 18–25 years, CS	SATAQ, FFTQ, PACS, EDI	Being at risk for an eating disorder, BMI, body-shape disstatisfaction were positively associated with each other.
Lebanon	Haddad *et al.*[27]	*N *= 811, Lebanese citizens, aged > 18 years, VS	EAT-26, BDS- EDI-2, HAM-D	At high risk for an eating disorder = 23.8%, being at high risk for an eating disorder was positively associated with depressive symptoms.
Lebanon^a^	Kanj *et al.*[46^▪^]	*N *= 403, Lebanese adolescents, aged 15–18 years, CS	EAT-7, BAS	Eating disorder pathology was associated with body-shape dissatisfaction and child abuse.
Lebanon^a^	Mina *et al.*[39]	*N *= 555, Lebanese citizens, aged 15- 18 years, VS	BES, RSES, BDS- EDI-2, PHQ-9, HAM-D	Binge-eating behavior was positively associated with body-shape dissatisfaction.
Lebanon^a^	Naamani *et al.*[32]	*N *= 129, Lebanese males who identified as homosexual, aged > 18 years, CS	EAT-26, MBDS	Eating disorder behaviors were not associated with body-shape dissatisfaction.
Lebanon	Rahme *et al.*[29]	*N *= 811, Lebanese citizens, aged > 18 years, VS	EES, BDS- EDI-2, RSES, HAM-D	Clinical levels of emotional eating = 32.4%, emotional eating more severe among adolescents compared to adults, emotional eating was positively associated with body-shape dissatisfaction and BMI.
Lebanon^a^	Sfeir *et al.*[33]	*N *= 811, Lebanese citizens, aged > 18 years, CS	BDS- EDI-2, DRES, BES, HAM-D, PSS	Binge-eating behavior and body-shape disstatisfaction were positively associated.
Lebanon^a^	Al-Musharaf *et al.*[40]	*N *= 555, Lebanese citizens, aged > 18 years, CS	BDS- EDI-2, RSES, PHQ-9, BDS	Body-shape dissatisfaction in eating disorders was positively associated with low self-esteem, anxiety, and stress.
Lebanon^a^	Khalil *et al.*[41]	*N *= 1001, Lebanese females, aged > 40 years, CS	BDS- EDI-2, DRES, BES, DOS, TOS	Eating disorder behaviors were positively associated with body-shape dissatisfaction.
Lebanon^a^	Gerges *et al.*[37^▪^]	*N *= 826, *N *= 555 adults, *N *= 433 pregnant women, Lebanese citizens, aged 15--18 years, CS	BDS- EDI-2, DEBQ	Women had greater levels of body-shape dissatisfaction compared to males.
Palestine/Israel^a^	Mansour *et al.*[34^▪^]	*N *= 361, Palestinian Arab and Israeli female university students, CS	EAT, EDI-2	Scores on eating disorder measures were similar between groups, westernization was positively associated with eating disorder pathology among Palestinian females in Haifa.
Saudi Arabia	Al-Shebali *et al.*[28]	*N *= 503, Saudi students, women, age range not mentioned, CS	EDDS, EDE-Q, BSQ, BFNE, PHQ-9, RSES, IWVS	At high risk for an eating disorder = 6.9%, at risk for bulimia nervosa = 4.4%, at risk for binge-eating disorder = 1.6%, at risk for other specified feeding or eating disorder = 1%, being at risk for an eating disorder was positively associated with westernization.
Saudi Arabia	Melisse *et al.*[26]	*N *= 2690, Saudi citizens, aged > 18 years, VS	EDE, EDE-Q	Score of two standard deviations above the average EDE score = 45%, at high risk for an eating disorder based on the EDE-Q = 28.8%, of which 28.5% women, and 29.7% men. EDE: objective binge-eating episodes = 4.1%, purging behavior = 0.0%. EDE-Q: objective binge-eating episodes = 5.9%, purging behavior = 2.1%.
Saudi Arabia	Melisse *et al.*[18^▪^]	*N *= 1225, Saudi citizens, aged > 18 years, CS	EDE-Q, BSQ, RSES, PSS, ARMSA, Media use, Socio-economic status	At high risk for an eating disorder based on the EDE-Q = 34.8%, 24.7% men, 36.3% women. At high risk for an eating disorder was positively associated with body-shape dissatisfaction and BMI. Single/ widowed/ separated marital status was associated with being at high risk for an eating disorder. Westernization, age, socio-economic status, self-esteem, perceived stress, and media use were not associated with being at a high risk for an eating disorder.
Saudi Arabia	Al Hadi *et al.*[25^▪▪^]	*N *= 4004, Saudi citizens, aged > 15 years, CS	WMH-CIDI 3.0	12-month eating disorder prevalence = 3.2%, 12-month prevalence of binge-eating disorder = 2.1%, bulimia nervosa = 1%. Eating disorder life-time prevalence = 6.1%, binge-eating disorder = 2.6%, bulimia nervosa = 2.8%, and anorexia nervosa = 0.6%. The onset of anorexia nervosa was at a younger age (*M* = 14.4 years), compared to bulimia nervosa (*M* = 19.7 years), and binge-eating disorder (*M* = 21.3 years).
Saudi Arabia	Melisse *et al.*[42]	*N *= 867, Saudi citizens, aged > 18 years	BSQ, EDE-SC	At high risk for clinical body-shape disstatisfaction as a symptom of an eating disorder = 26.7%.
Oman	Divecha *et al.*[29]	*N *= 351, Omani Medical college students, CS	FRS	Dissatisfied with body-shape = 80%.

BDS- EDI-2, Body Dissatisfaction subscale of the Eating Disorder Inventory 2; BDS, Beirut Distress Scale; BES, Binge Eating Scale; BFNE, Brief version of the Fear of Negative Evaluation scale; BIG, Bodybuilder Image Grid; BSQ, Body Shape Questionnaire; CS, Cross- Sectional; DEBQ, Dutch Eating Behavior Questionnaire; DOS, Dusseldorf Orthorexia Scale; DRES, Dutch Restrained Eating Scale; EAT, Eating Attitude Test; EDDS, Eating Disorder Diagnostic Scale; EDE, Eating Disorder Examination; EDE-Q, Eating Disorder Examination- Questionnaire; EES, Emotional Eating Scale; FFTQ, Fat Talk Questionnaire; FRS, Figure Rating Scale; HAM-D, Hamilton Depression Rating Scale; IWVS, Internalization of Western Values Scale; MBDS, Male Body Dissatisfaction Scale; PACS, Physical Appearance Comparison Scale; PHQ 9, Patient Health Questionnaire; PSS, Perceived Stress Scale; RSES, Rosenberg Self-Esteem Scale; SATAQ, Sociocultural Attitudes Towards Appearance Questionnaire; TOS, Teruel Orthorexia Scale; VS, Validation Study; WMH-CIDI 3.0, WHO Composite International Diagnostic Interview.

a Number of participants that scored above the clinical cutoff not mentioned.

### Eating disorders

Table 1 summarizes that two studies used interview data to examine the prevalence of eating disorders in Saudi Arabia (see Table 1). One of them was a national health survey, which reported a 12-month eating disorder prevalence of 3.2%, of which BED was the most prevalent eating disorder (2.1%). Eating disorder lifetime prevalence was 6.1%, of which BED (2.6%) was the most prevalent eating disorder [25^▪▪^]. In addition, in another study, 45% of the participants scored 2 standard deviations above average, suggesting a high prevalence of eating problems. However, it should be noted that the sample was biased, as mainly high educated women, and men and women with an interest in healthcare were interviewed [26].

### At high risk for eating disorders

As reported in Table 1, based on eating disorder self-report assessment tools, 23.8–34.8% scored above the local clinical cutoff and were therefore at high risk for an eating disorder (women: 28.5–36.3%, men: 24.7–29.7%) [18^▪^,^26^,^27^]. In addition, 6.9% of the female students were at high risk for an eating disorder, of which 4.4% for bulimia nervosa, 1.6% for BED and 1% for other specified feeding or eating disorder [28]. Furthermore, 26.7% of mainly highly educated young girls scored above the clinical cutoff on the Body Shape Questionnaire, which assessed body-shape dissatisfaction as a symptom of an eating disorder [18^▪^], and 80% of medical college students reported on the Figure Rating Scale to be dissatisfied with their body-shape [29].

### Eating disorder behaviors

Table 1 summarizes that clinical levels of emotional eating were reported by 32.4% [30], and 20.2% of the adolescents and young adults reported binge eating [31^▪^]. More specifically, 4.1–5.9% had at least one objective binge-eating episode per week. In addition, daily binge-eating episodes were reported by 2.6%, and purging behavior by 0.2% [26].

### Correlates of eating disorders, at high risk for eating disorders and eating disorder behaviors

Table 1 summarizes the various correlates of eating disorder pathology.

#### BMI and body-shape dissatisfaction

Lifetime eating disorder prevalence of bulimia nervosa and BED, being at risk for an eating disorder, eating disorder behaviors, and body-shape dissatisfaction were positively associated with BMI [18^▪^,^25^^▪▪^,^26^]. Conversely, the association between eating disorder behaviors and body-shape dissatisfaction was not confirmed among homosexually oriented males [32].

#### Comorbid psychopathology and a low self-esteem

Lifetime eating disorder prevalence, 12-month eating disorder prevalence, being at risk for an eating disorder, and binge eating were associated with comorbid psychopathology such as anxiety, mood, and impulse-control disorders [25^▪▪^,^27^,31^▪^]. Being at high risk for an eating disorder, and eating disorder behavior were not associated with a low self-esteem [18^▪^,^30^,^33^].

#### Age

Age was negatively associated with eating disorder pathology: adolescents had more frequent eating disorder behaviors in comparison with adults [29]. However, another study found that the type of eating disorder diagnosis was associated with age: the onset of anorexia nervosa was at a younger age (*M* = 14.4 years), compared to bulimia nervosa (*M* = 19.7 years), and BED (*M* = 21.3 years) [25^▪▪^].

#### Socioeconomic status

Some studies concluded that being at high risk for an eating disorder was associated with a higher socioeconomic status [28,34^▪^]. However, not all studies included in the present review confirmed this association [18^▪^]. Furthermore, some studies found that lifetime, as well as 12-month eating disorder prevalence [25^▪▪^], and binge-eating behavior [33] were associated with a higher level of education, while other studies were unable to confirm these associations [18^▪^,^26^].

#### Marital status and westernization

Lifetime eating disorder prevalence, 12-month eating disorder prevalence [25^▪▪^], and being at high risk for an eating disorder [18^▪^] and binge-eating behavior [33] were associated with a separated/widowed, or single marital status. Some studies concluded that being at high risk for an eating disorder was associated with westernization [28,34^▪^]. However, not all studies included in the present review confirmed this association [18^▪^].

## CONCLUSION

The aim of the present systematic review was to provide an update regarding the epidemiology of eating disorders in the Arab world. The 12-month eating disorder prevalence was 3.2%, and eating disorder life-time prevalence was 6.1%. BED was the most common eating disorder (12-month prevalence = 2.1%, lifetime prevalence = 2.6%, at high risk = 1.6%), binge-eating behavior was the most prevalent eating disorder behavior. In addition, between 23.8 and 34.8% of the population under study was at high risk for an eating disorder. Conversely, the systematic review published in 2020 [9] found larger variations in the range of the population being at high risk for an eating disorder. Among men, between 2 and 41% had a score above a clinical cutoff on an eating disorder self-report measure, and was therefore at a high risk for an eating disorder. Among women, 11–55% was at high risk (overall mean = 32.3%, overall median 32.5%). Furthermore, none of the studies did use validated assessment tools and norms appropriate for the culture at hand, while the majority of studies included in the present systematic review used validated assessment tools, and some used appropriate norms for the culture at hand. In light of the present systematic review, it can be inferred with greater confidence that approximately 30% of the total population is at high risk for an eating disorder. In both systematic reviews, a high BMI and body-shape dissatisfaction, as well as a separated, widowed, or single status were positively associated with eating disorder pathology.

It should be acknowledged that several studies included in the present review conducted in Lebanon originated from two distinct datasets, including *N* = 811 [29,33,35,36,37^▪^], and *N* = 555 [38–41] participants. This may have implications for the generalizability of the results. In addition, there is a lack of studies conducted in the Arab world. The present study was unable to identify studies conducted among clinical samples, and no studies were identified conducted in Jordan Kuwait, the UAE, or Qatar. However, compared to 2020 [9], in the realm of eating disorder, research advances are being made: culture sensitive norms for self-reports are available [26,42], eating disorders are examined in national health surveys [25^▪▪^], and a pilot trial of the Body Project an eating disorder preventive program addressing body-shape dissatisfaction was conducted (54). Conversely, for a long time, there is a lack of specialized eating disorder therapists and a gap between treatment supply and demand [43]. To date, only two mental healthcare centers, both located in the UAE, offer specialized multidisciplinary eating disorder treatment in the Gulf area (el Khazen C, personal communication, 28 December 2023). There is an urgent need to increase access to specialized care for eating disorders in the Arab world. Subsequently, it is recommended to implement preventive programs.

## Acknowledgements

*The manuscript has been written by B.M. in collaboration with H.W.H. and E.vF.*

### Financial support and sponsorship

*None.*

### Conflicts of interest

*There are no conflicting interests.*

## Supplementary Material

Supplemental Digital Content

## Supplementary Material

Supplemental Digital Content

## REFERENCES AND RECOMMENDED READING

Papers of particular interest, published within the annual period of review, have been highlighted as:

▪ of special interest
▪▪ of outstanding interest
